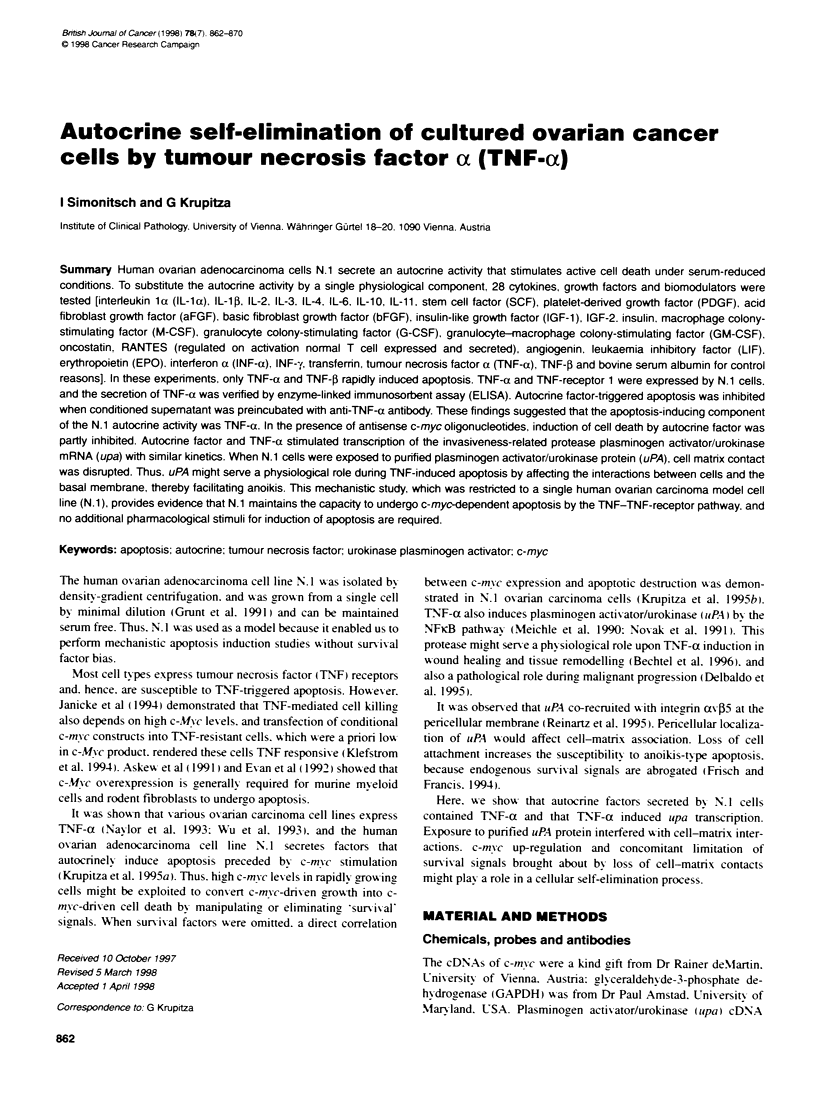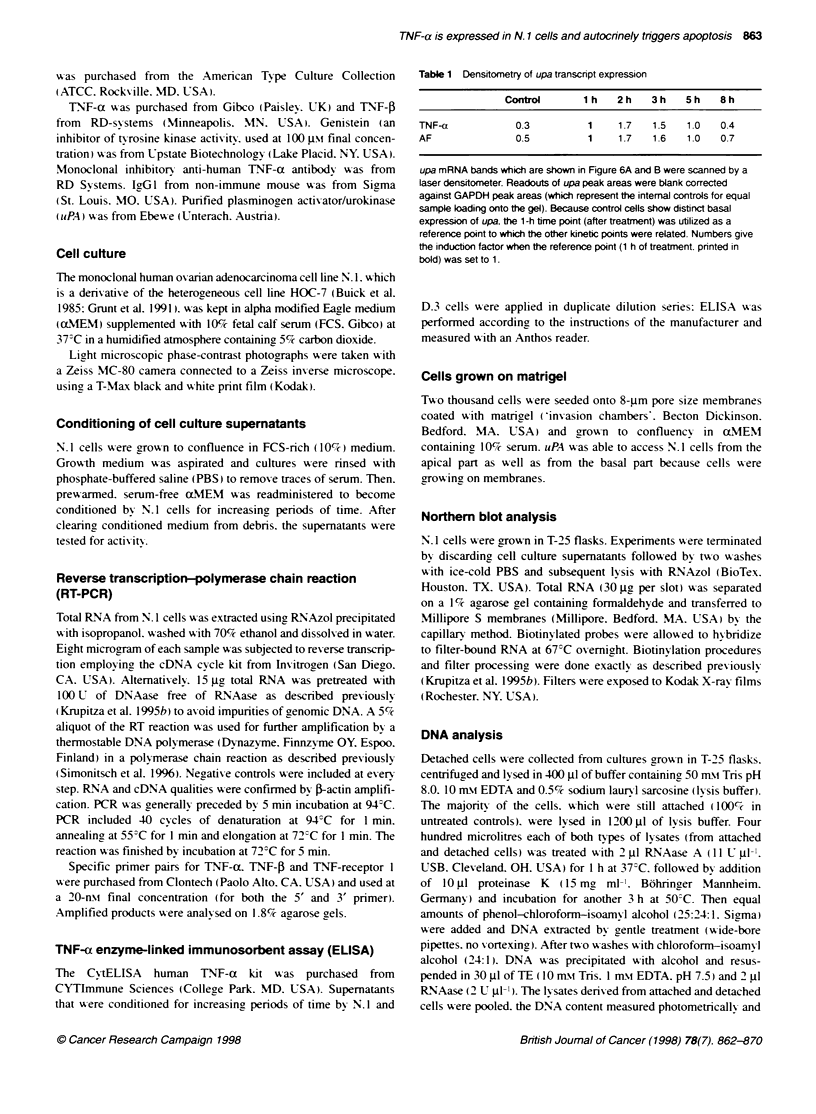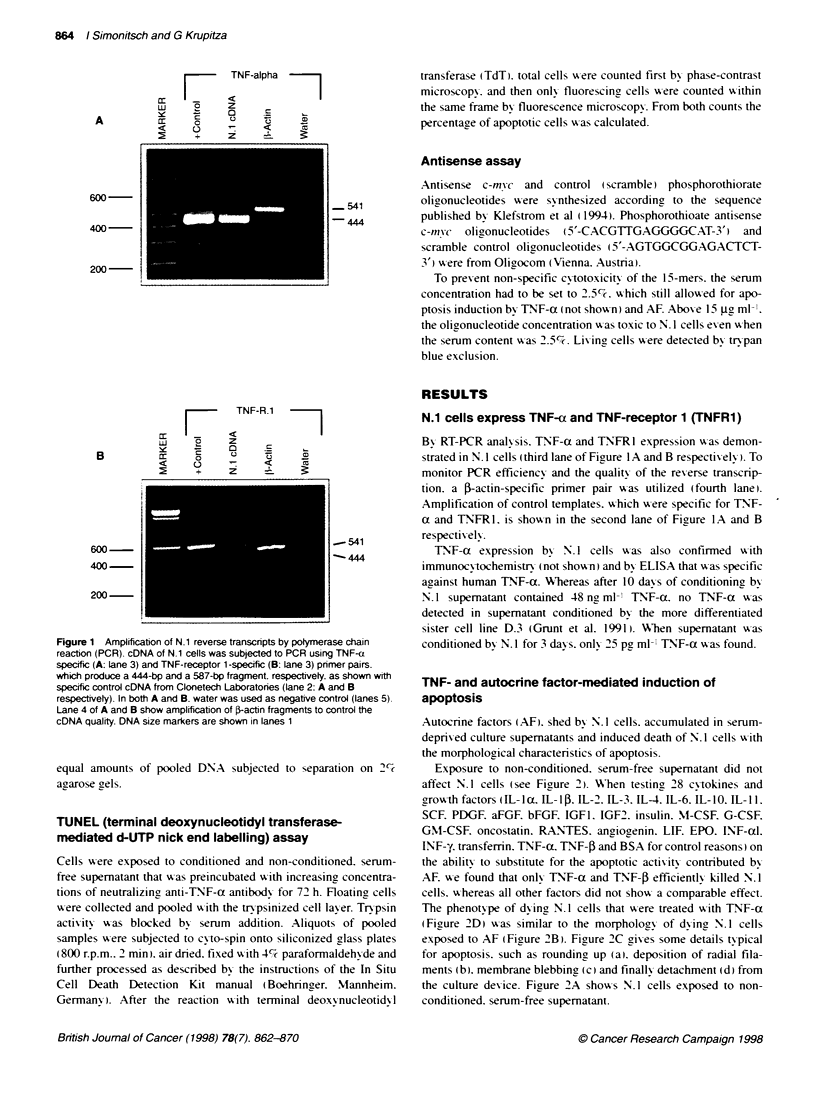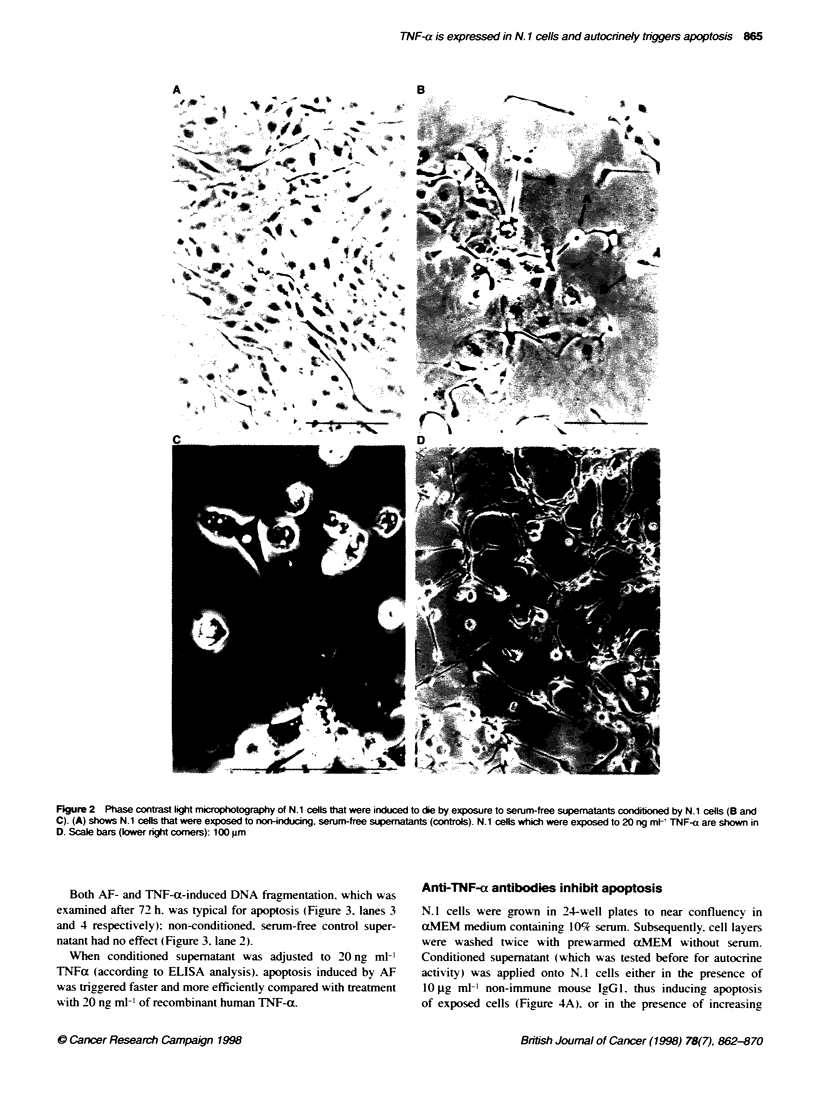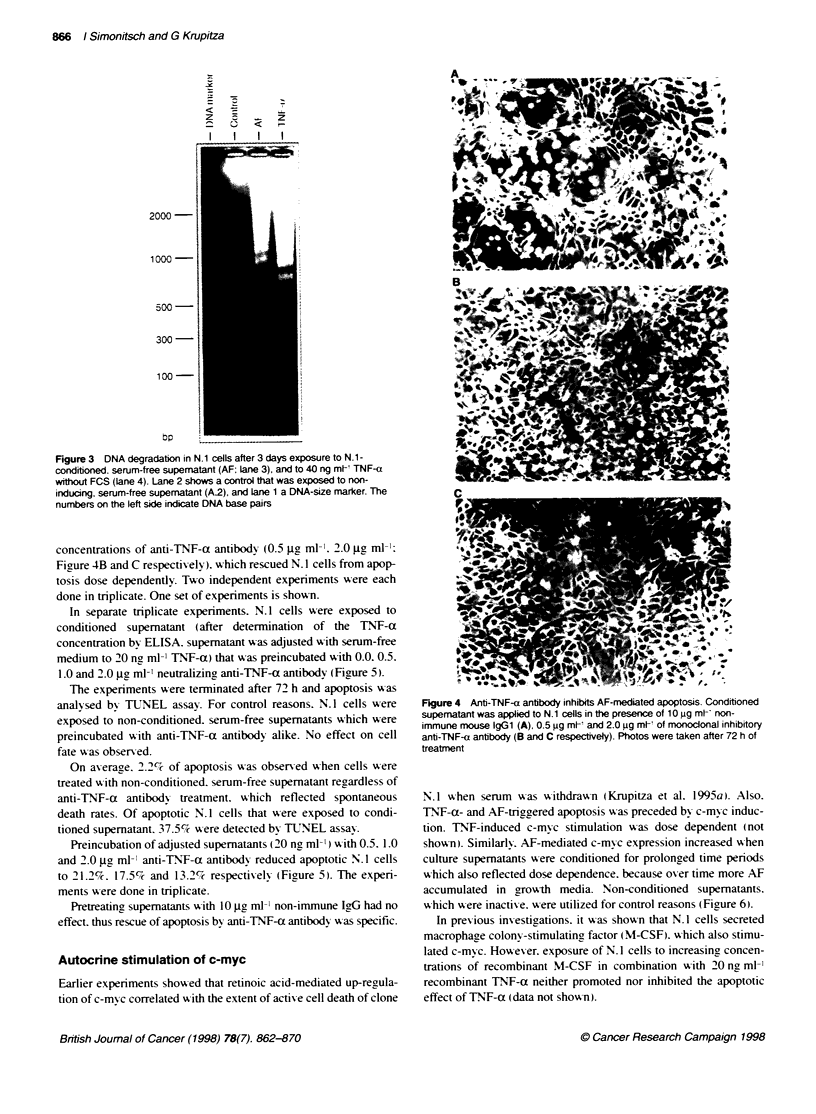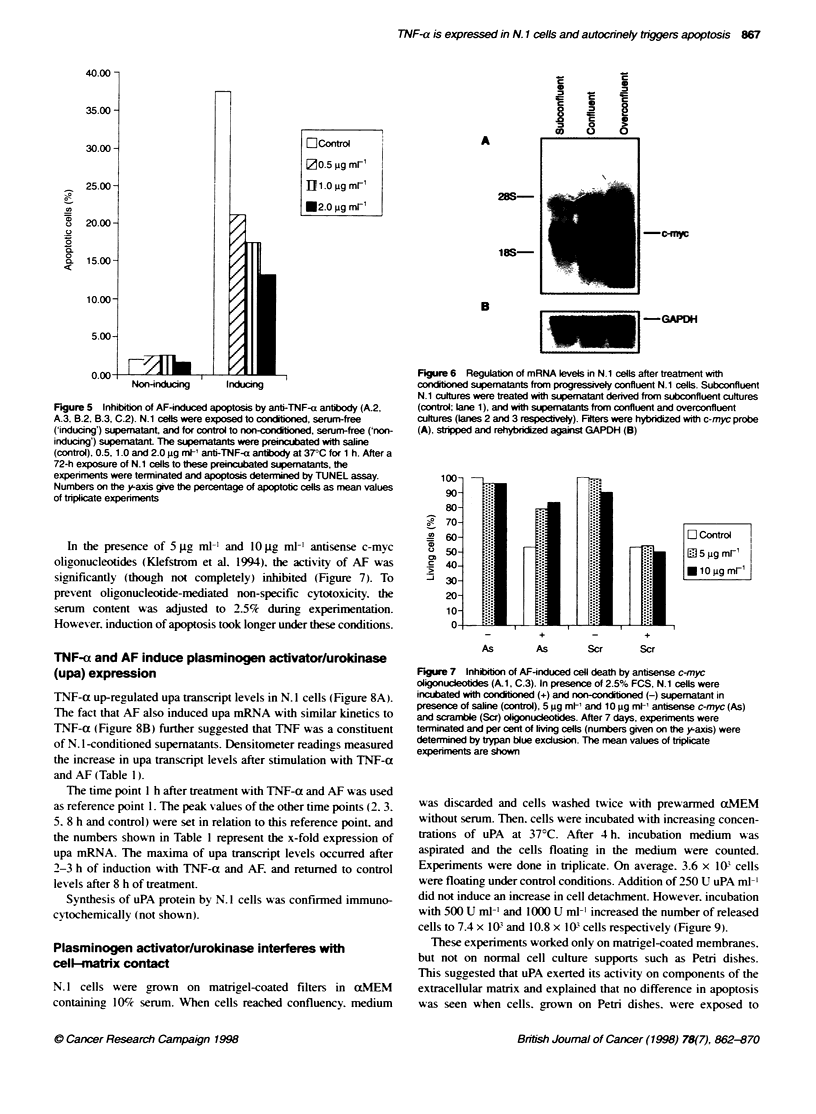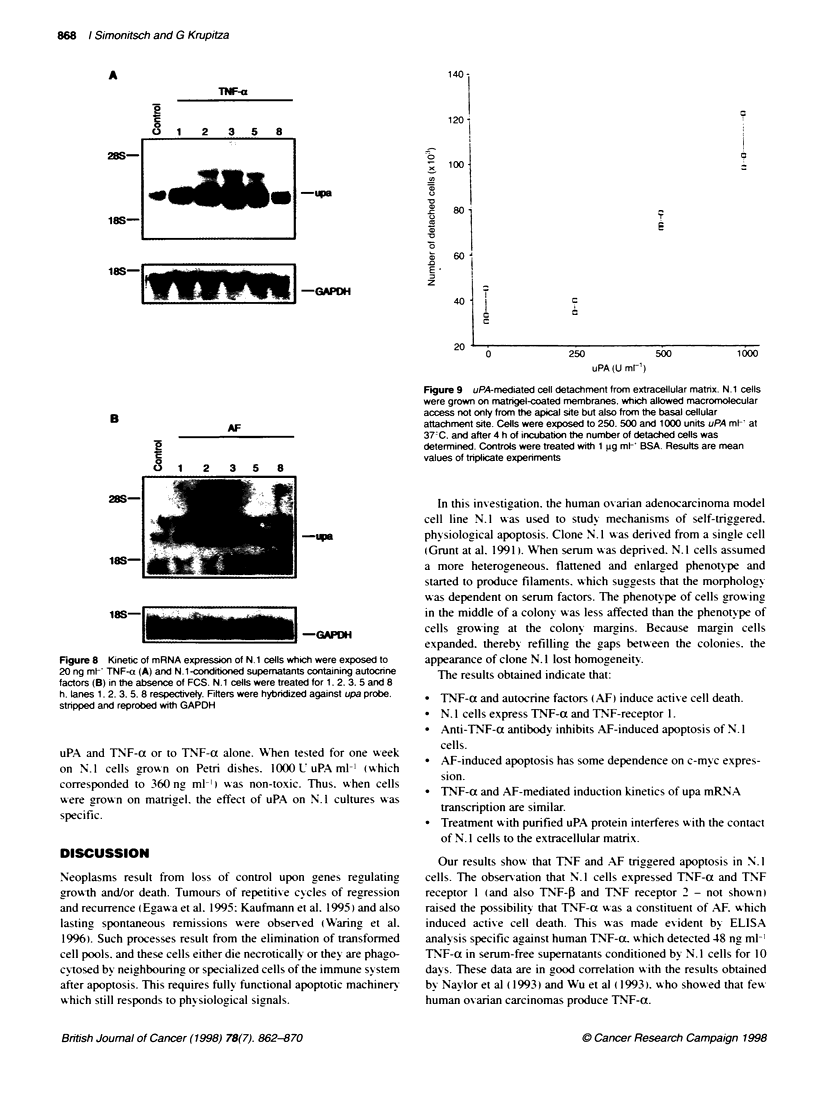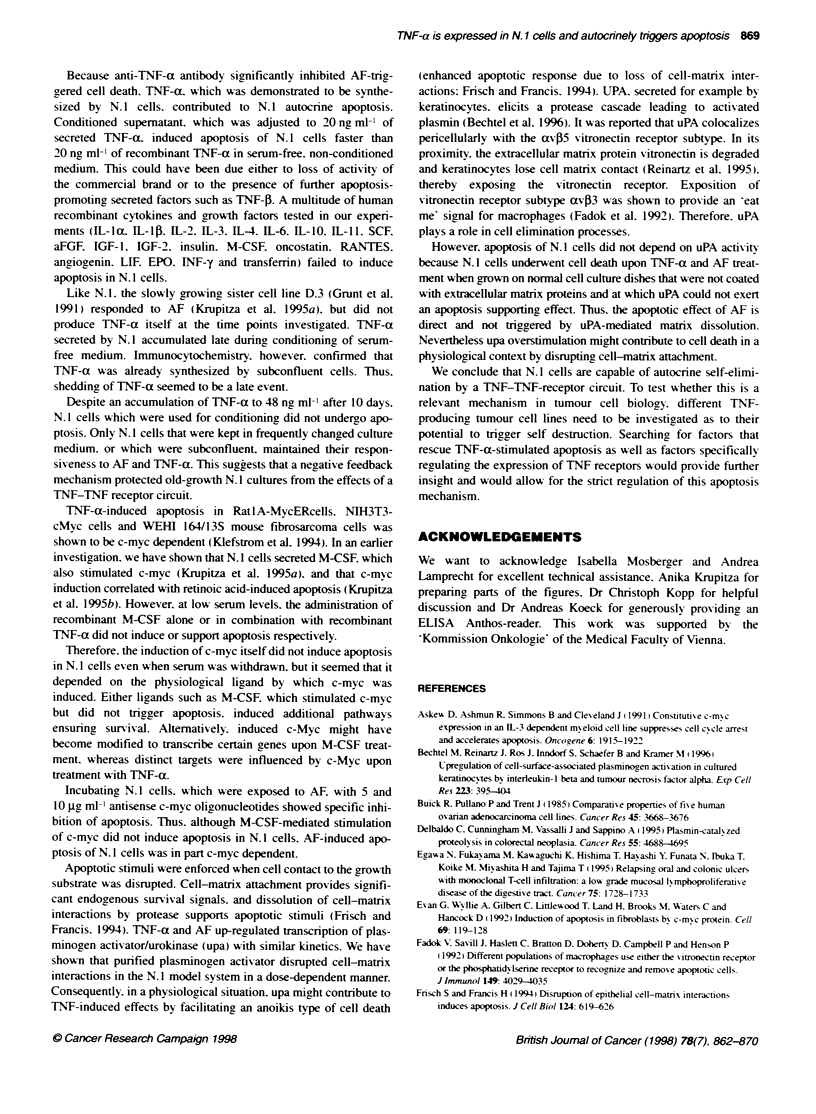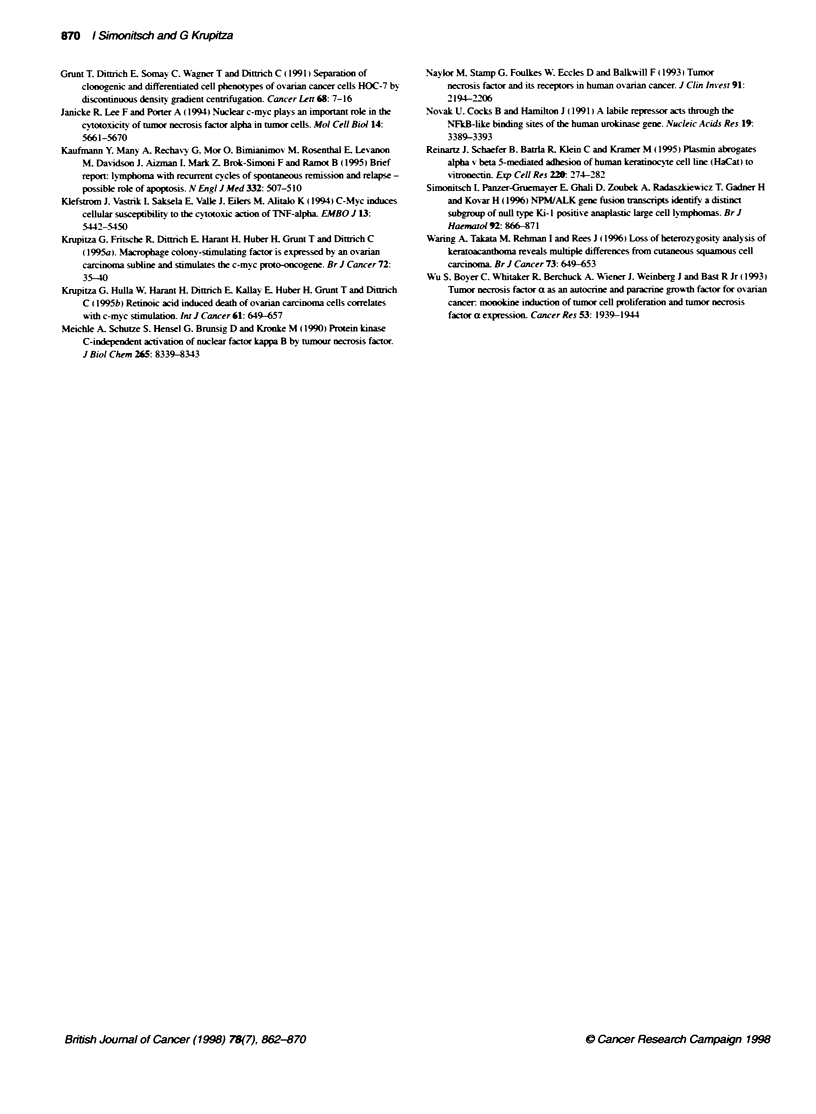# Autocrine self-elimination of cultured ovarian cancer cells by tumour necrosis factor alpha (TNF-alpha).

**DOI:** 10.1038/bjc.1998.594

**Published:** 1998-10

**Authors:** I. Simonitsch, G. Krupitza

**Affiliations:** Institute of Clinical Pathology, University of Vienna, Austria.

## Abstract

**Images:**


British Journal of Cancer (1998) 78(7), 862–870
© 1998 Cancer Research Campaign

### Autocrine self-elimination of cultured ovarian cancer cells by tumour necrosis factor α (TNF-α)

**I Simonitsch and G Krupitza**

Institute of Clinical Pathology, University of Vienna, Währinger Gürtel 18–20, 1090 Vienna, Austria

**Summary** Human ovarian adenocarcinoma cells N.1 secrete an autocrine activity that stimulates active cell death under serum-reduced conditions. To substitute the autocrine activity by a single physiological component, 28 cytokines, growth factors and biomodulators were tested [interleukin 1α (IL-1α), IL-1β, IL-2, IL-3, IL-4, IL-6, IL-10, IL-11, stem cell factor (SCF), platelet-derived growth factor (PDGF), acid fibroblast growth factor (aFGF), basic fibroblast growth factor (bFGF), insulin-like growth factor (IGF-1), IGF-2, insulin, macrophage colony-stimulating factor (M-CSF), granulocyte colony-stimulating factor (G-CSF), granulocyte–macrophage colony-stimulating factor (GM-CSF), oncostatin, RANTES (regulated on activation normal T cell expressed and secreted), angiogenin, leukaemia inhibitory factor (LIF), erythropoietin (EPO), interferon α (INF-α), INF-γ, transferrin, tumour necrosis factor α (TNF-α), TNF-β and bovine serum albumin for control reasons]. In these experiments, only TNF-α and TNF-β rapidly induced apoptosis. TNF-α and TNF-receptor 1 were expressed by N.1 cells, and the secretion of TNF-α was verified by enzyme-linked immunosorbent assay (ELISA). Autocrine factor-triggered apoptosis was inhibited when conditioned supernatant was preincubated with anti-TNF-α antibody. These findings suggested that the apoptosis-inducing component of the N.1 autocrine activity was TNF-α. In the presence of antisense *c-myc* oligonucleotides, induction of cell death by autocrine factor was partly inhibited. Autocrine factor and TNF-α stimulated transcription of the invasiveness-related protease plasminogen activator/urokinase mRNA (*upa*) with similar kinetics. When N.1 cells were exposed to purified plasminogen activator/urokinase protein (*uPA*), cell matrix contact was disrupted. Thus, *uPA* might serve a physiological role during TNF-induced apoptosis by affecting the interactions between cells and the basal membrane, thereby facilitating anoikis. This mechanistic study, which was restricted to a single human ovarian carcinoma model cell line (N.1), provides evidence that N.1 maintains the capacity to undergo *c-myc*-dependent apoptosis by the TNF–TNF-receptor pathway, and no additional pharmacological stimuli for induction of apoptosis are required.

**Keywords:** apoptosis; autocrine; tumour necrosis factor; urokinase plasminogen activator; *c-myc*

The human ovarian adenocarcinoma cell line N.1 was isolated by density-gradient centrifugation, and was grown from a single cell by minimal dilution (Grunt et al, 1991) and can be maintained serum free. Thus, N.1 was used as a model because it enabled us to perform mechanistic apoptosis induction studies without survival factor bias.

Most cell types express tumour necrosis factor (TNF) receptors and, hence, are susceptible to TNF-triggered apoptosis. However, Janicke et al (1994) demonstrated that TNF-mediated cell killing also depends on high *c-Myc* levels, and transfection of conditional *c-myc* constructs into TNF-resistant cells, which were a priori low in *c-Myc* product, rendered these cells TNF responsive (Klefstrom et al, 1994). Askew et al (1991) and Evan et al (1992) showed that *c-Myc* overexpression is generally required for murine myeloid cells and rodent fibroblasts to undergo apoptosis.

It was shown that various ovarian carcinoma cell lines express TNF-α (Naylor et al, 1993; Wu et al, 1993), and the human ovarian adenocarcinoma cell line N.1 secretes factors that autocrinely induce apoptosis preceded by *c-myc* stimulation (Krupitza et al, 1995*a*). Thus, high *c-myc* levels in rapidly growing cells might be exploited to convert *c-myc*-driven growth into *c-myc*-driven cell death by manipulating or eliminating 'survival' signals. When survival factors were omitted, a direct correlation between *c-myc* expression and apoptotic destruction was demonstrated in N.1 ovarian carcinoma cells (Krupitza et al, 1995*b*). TNF-α also induces plasminogen activator/urokinase (*uPA*) by the NFκB pathway (Meichle et al, 1990; Novak et al, 1991). This protease might serve a physiological role upon TNF-α induction in wound healing and tissue remodelling (Bechtel et al, 1996), and also a pathological role during malignant progression (Delbaldo et al, 1995).

It was observed that *uPA* co-recruited with integrin αvβ5 at the pericellular membrane (Reinartz et al, 1995). Pericellular localization of *uPA* would affect cell–matrix association. Loss of cell attachment increases the susceptibility to anoikis-type apoptosis, because endogenous survival signals are abrogated (Frisch and Francis, 1994).

Here, we show that autocrine factors secreted by N.1 cells contained TNF-α and that TNF-α induced *upa* transcription. Exposure to purified *uPA* protein interfered with cell–matrix interactions. *c-myc* up-regulation and concomitant limitation of survival signals brought about by loss of cell–matrix contacts might play a role in a cellular self-elimination process.

*Received 10 October 1997*
*Revised 5 March 1998*
*Accepted 1 April 1998*

*Correspondence to:* G Krupitza

### MATERIAL AND METHODS

### Chemicals, probes and antibodies

The cDNAs of *c-myc* were a kind gift from Dr Rainer deMartin, University of Vienna, Austria; glyceraldehyde-3-phosphate dehydrogenase (GAPDH) was from Dr Paul Amstad, University of Maryland, USA. Plasminogen activator/urokinase (*upa*) cDNA

was purchased from the American Type Culture Collection (ATCC, Rockville, MD, USA).

TNF-α was purchased from Gibco (Paisley, UK) and TNF-β from RD-systems (Minneapolis, MN, USA). Genistein (an inhibitor of tyrosine kinase activity, used at 100 μM final concentration) was from Upstate Biotechnology (Lake Placid, NY, USA). Monoclonal inhibitory anti-human TNF-α antibody was from RD Systems. IgG1 from non-immune mouse was from Sigma (St. Louis, MO, USA). Purified plasminogen activator/urokinase (*uPA*) was from Ebewe (Unterach, Austria).

### Cell culture

The monoclonal human ovarian adenocarcinoma cell line N.1, which is a derivative of the heterogeneous cell line HOC-7 (Buick et al, 1985; Grunt et al, 1991), was kept in alpha modified Eagle medium (αMEM) supplemented with 10% fetal calf serum (FCS, Gibco) at 37°C in a humidified atmosphere containing 5% carbon dioxide.

Light microscopic phase-contrast photographs were taken with a Zeiss MC-80 camera connected to a Zeiss inverse microscope, using a T-Max black and white print film (Kodak).

### Conditioning of cell culture supernatants

N.1 cells were grown to confluence in FCS-rich (10%) medium. Growth medium was aspirated and cultures were rinsed with phosphate-buffered saline (PBS) to remove traces of serum. Then, prewarmed, serum-free αMEM was readministered to become conditioned by N.1 cells for increasing periods of time. After clearing conditioned medium from debris, the supernatants were tested for activity.

### Reverse transcription–polymerase chain reaction (RT-PCR)

Total RNA from N.1 cells was extracted using RNAzol precipitated with isopropanol, washed with 70% ethanol and dissolved in water. Eight microgram of each sample was subjected to reverse transcription employing the cDNA cycle kit from Invitrogen (San Diego, CA, USA). Alternatively, 15 μg total RNA was pretreated with 100 U of DNAase free of RNAase as described previously (Krupitza et al, 1995*b*) to avoid impurities of genomic DNA. A 5% aliquot of the RT reaction was used for further amplification by a thermostable DNA polymerase (Dynazyme, Finnzyme OY, Espoo, Finland) in a polymerase chain reaction as described previously (Simonitsch et al, 1996). Negative controls were included at every step. RNA and cDNA qualities were confirmed by β-actin amplification. PCR was generally preceded by 5 min incubation at 94°C. PCR included 40 cycles of denaturation at 94°C for 1 min, annealing at 55°C for 1 min and elongation at 72°C for 1 min. The reaction was finished by incubation at 72°C for 5 min.

Specific primer pairs for TNF-α, TNF-β and TNF-receptor 1 were purchased from Clontech (Paolo Alto, CA, USA) and used at a 20-nM final concentration (for both the 5′ and 3′ primer). Amplified products were analysed on 1.8% agarose gels.

### TNF-α enzyme-linked immunosorbent assay (ELISA)

The CytELISA human TNF-α kit was purchased from CYTImmune Sciences (College Park, MD, USA). Supernatants that were conditioned for increasing periods of time by N.1 and D.3 cells were applied in duplicate dilution series; ELISA was performed according to the instructions of the manufacturer and measured with an Anthos reader.

**Table 1** Densitometry of *upa* transcript expression

| | Control | 1 h | 2 h | 3 h | 5 h | 8 h |
|---|---|---|---|---|---|---|
| TNF-α | 0.3 | **1** | 1.7 | 1.5 | 1.0 | 0.4 |
| AF | 0.5 | **1** | 1.7 | 1.6 | 1.0 | 0.7 |

*upa* mRNA bands which are shown in Figure 6A and B were scanned by a laser densitometer. Readouts of *upa* peak areas were blank corrected against GAPDH peak areas (which represent the internal controls for equal sample loading onto the gel). Because control cells show distinct basal expression of *upa*, the 1-h time point (after treatment) was utilized as a reference point to which the other kinetic points were related. Numbers give the induction factor when the reference point (1 h of treatment, printed in bold) was set to 1.

### Cells grown on matrigel

Two thousand cells were seeded onto 8-μm pore size membranes coated with matrigel ('invasion chambers', Becton Dickinson, Bedford, MA, USA) and grown to confluency in αMEM containing 10% serum. *uPA* was able to access N.1 cells from the apical part as well as from the basal part because cells were growing on membranes.

### Northern blot analysis

N.1 cells were grown in T-25 flasks. Experiments were terminated by discarding cell culture supernatants followed by two washes with ice-cold PBS and subsequent lysis with RNAzol (BioTex, Houston, TX, USA). Total RNA (30 μg per slot) was separated on a 1% agarose gel containing formaldehyde and transferred to Millipore S membranes (Millipore, Bedford, MA, USA) by the capillary method. Biotinylated probes were allowed to hybridize to filter-bound RNA at 67°C overnight. Biotinylation procedures and filter processing were done exactly as described previously (Krupitza et al, 1995*b*). Filters were exposed to Kodak X-ray films (Rochester, NY, USA).

### DNA analysis

Detached cells were collected from cultures grown in T-25 flasks, centrifuged and lysed in 400 μl of buffer containing 50 mM Tris pH 8.0, 10 mM EDTA and 0.5% sodium lauryl sarcosine (lysis buffer). The majority of the cells, which were still attached (100% in untreated controls), were lysed in 1200 μl of lysis buffer. Four hundred microlitres each of both types of lysates (from attached and detached cells) was treated with 2 μl RNAase A (11 U $\mu l^{-1}$, USB, Cleveland, OH, USA) for 1 h at 37°C, followed by addition of 10 μl proteinase K (15 mg $ml^{-1}$, Böhringer Mannheim, Germany) and incubation for another 3 h at 50°C. Then equal amounts of phenol–chloroform–isoamyl alcohol (25:24:1, Sigma) were added and DNA extracted by gentle treatment (wide-bore pipettes, no vortexing). After two washes with chloroform–isoamyl alcohol (24:1), DNA was precipitated with alcohol and resuspended in 30 μl of TE (10 mM Tris, 1 mM EDTA, pH 7.5) and 2 μl RNAase (2 U $\mu l^{-1}$). The lysates derived from attached and detached cells were pooled, the DNA content measured photometrically and

© Cancer Research Campaign 1998

**Figure 1** Amplification of N.1 reverse transcripts by polymerase chain reaction (PCR). cDNA of N.1 cells was subjected to PCR using TNF-α specific (**A**: lane 3) and TNF-receptor 1-specific (**B**: lane 3) primer pairs, which produce a 444-bp and a 587-bp fragment, respectively, as shown with specific control cDNA from Clonetech Laboratories (lane 2: **A** and **B** respectively). In both **A** and **B**, water was used as negative control (lanes 5). Lane 4 of **A** and **B** show amplification of β-actin fragments to control the cDNA quality. DNA size markers are shown in lanes 1

equal amounts of pooled DNA subjected to separation on 2% agarose gels.

### TUNEL (terminal deoxynucleotidyl transferase-mediated d-UTP nick end labelling) assay

Cells were exposed to conditioned and non-conditioned, serum-free supernatant that was preincubated with increasing concentrations of neutralizing anti-TNF-α antibody for 72 h. Floating cells were collected and pooled with the trypsinized cell layer. Trypsin activity was blocked by serum addition. Aliquots of pooled samples were subjected to cyto-spin onto siliconized glass plates (800 r.p.m., 2 min), air dried, fixed with 4% paraformaldehyde and further processed as described by the instructions of the In Situ Cell Death Detection Kit manual (Boehringer, Mannheim, Germany). After the reaction with terminal deoxynucleotidyl transferase (TdT), total cells were counted first by phase-contrast microscopy, and then only fluorescing cells were counted within the same frame by fluorescence microscopy. From both counts the percentage of apoptotic cells was calculated.

### Antisense assay

Antisense c-*myc* and control (scramble) phosphorothiorate oligonucleotides were synthesized according to the sequence published by Klefstrom et al (1994). Phosphorothioate antisense c-*myc* oligonucleotides (5′-CACGTTGAGGGGCAT-3′) and scramble control oligonucleotides (5′-AGTGGCGGAGACTCT-3′) were from Oligocom (Vienna, Austria).

To prevent non-specific cytotoxicity of the 15-mers, the serum concentration had to be set to 2.5%, which still allowed for apoptosis induction by TNF-α (not shown) and AF. Above 15 μg $ml^{-1}$, the oligonucleotide concentration was toxic to N.1 cells even when the serum content was 2.5%. Living cells were detected by trypan blue exclusion.

### RESULTS

### N.1 cells express TNF-α and TNF-receptor 1 (TNFR1)

By RT-PCR analysis, TNF-α and TNFR1 expression was demonstrated in N.1 cells (third lane of Figure 1A and B respectively). To monitor PCR efficiency and the quality of the reverse transcription, a β-actin-specific primer pair was utilized (fourth lane). Amplification of control templates, which were specific for TNF-α and TNFR1, is shown in the second lane of Figure 1A and B respectively.

TNF-α expression by N.1 cells was also confirmed with immunocytochemistry (not shown) and by ELISA that was specific against human TNF-α. Whereas after 10 days of conditioning by N.1 supernatant contained 48 ng $ml^{-1}$ TNF-α, no TNF-α was detected in supernatant conditioned by the more differentiated sister cell line D.3 (Grunt et al, 1991). When supernatant was conditioned by N.1 for 3 days, only 25 pg $ml^{-1}$ TNF-α was found.

### TNF- and autocrine factor-mediated induction of apoptosis

Autocrine factors (AF), shed by N.1 cells, accumulated in serum-deprived culture supernatants and induced death of N.1 cells with the morphological characteristics of apoptosis.

Exposure to non-conditioned, serum-free supernatant did not affect N.1 cells (see Figure 2). When testing 28 cytokines and growth factors (IL-1α, IL-1β, IL-2, IL-3, IL-4, IL-6, IL-10, IL-11, SCF, PDGF, aFGF, bFGF, IGF1, IGF2, insulin, M-CSF, G-CSF, GM-CSF, oncostatin, RANTES, angiogenin, LIF, EPO, INF-αl, INF-γ, transferrin, TNF-α, TNF-β and BSA for control reasons) on the ability to substitute for the apoptotic activity contributed by AF, we found that only TNF-α and TNF-β efficiently killed N.1 cells, whereas all other factors did not show a comparable effect. The phenotype of dying N.1 cells that were treated with TNF-α (Figure 2D) was similar to the morphology of dying N.1 cells exposed to AF (Figure 2B). Figure 2C gives some details typical for apoptosis, such as rounding up (a), deposition of radial filaments (b), membrane blebbing (c) and finally detachment (d) from the culture device. Figure 2A shows N.1 cells exposed to non-conditioned, serum-free supernatant.

© Cancer Research Campaign 1998

**Figure 2** Phase contrast light microphotography of N.1 cells that were induced to die by exposure to serum-free supernatants conditioned by N.1 cells (**B** and **C**). (**A**) shows N.1 cells that were exposed to non-inducing, serum-free supernatants (controls). N.1 cells which were exposed to 20 ng ml$^{-1}$ TNF-α are shown in **D**. Scale bars (lower right corners): 100 μm

Both AF- and TNF-α-induced DNA fragmentation, which was examined after 72 h, was typical for apoptosis (Figure 3, lanes 3 and 4 respectively): non-conditioned, serum-free control supernatant had no effect (Figure 3, lane 2).

When conditioned supernatant was adjusted to 20 ng ml$^{-1}$ TNFα (according to ELISA analysis), apoptosis induced by AF was triggered faster and more efficiently compared with treatment with 20 ng ml$^{-1}$ of recombinant human TNF-α.

### Anti-TNF-α antibodies inhibit apoptosis

N.1 cells were grown in 24-well plates to near confluency in αMEM medium containing 10% serum. Subsequently, cell layers were washed twice with prewarmed αMEM without serum. Conditioned supernatant (which was tested before for autocrine activity) was applied onto N.1 cells either in the presence of 10 μg ml$^{-1}$ non-immune mouse IgG1, thus inducing apoptosis of exposed cells (Figure 4A), or in the presence of increasing

© Cancer Research Campaign 1998 

**Figure 3** DNA degradation in N.1 cells after 3 days exposure to N.1-conditioned, serum-free supernatant (AF; lane 3), and to 40 ng ml$^{-1}$ TNF-α without FCS (lane 4). Lane 2 shows a control that was exposed to non-inducing, serum-free supernatant (A.2), and lane 1 a DNA-size marker. The numbers on the left side indicate DNA base pairs concentrations of anti-TNF-α antibody (0.5 μg ml$^{-1}$, 2.0 μg ml$^{-1}$; Figure 4B and C respectively), which rescued N.1 cells from apoptosis dose dependently. Two independent experiments were each done in triplicate. One set of experiments is shown.

In separate triplicate experiments, N.1 cells were exposed to conditioned supernatant (after determination of the TNF-α concentration by ELISA, supernatant was adjusted with serum-free medium to 20 ng ml$^{-1}$ TNF-α) that was preincubated with 0.0, 0.5, 1.0 and 2.0 μg ml$^{-1}$ neutralizing anti-TNF-α antibody (Figure 5).

The experiments were terminated after 72 h and apoptosis was analysed by TUNEL assay. For control reasons, N.1 cells were exposed to non-conditioned, serum-free supernatants which were preincubated with anti-TNF-α antibody alike. No effect on cell fate was observed.

On average, 2.2% of apoptosis was observed when cells were treated with non-conditioned, serum-free supernatant regardless of anti-TNF-α antibody treatment, which reflected spontaneous death rates. Of apoptotic N.1 cells that were exposed to conditioned supernatant, 37.5% were detected by TUNEL assay.

Preincubation of adjusted supernatants (20 ng ml$^{-1}$) with 0.5, 1.0 and 2.0 μg ml$^{-1}$ anti-TNF-α antibody reduced apoptotic N.1 cells to 21.2%, 17.5% and 13.2% respectively (Figure 5). The experiments were done in triplicate.

Pretreating supernatants with 10 μg ml$^{-1}$ non-immune IgG had no effect, thus rescue of apoptosis by anti-TNF-α antibody was specific.

### Autocrine stimulation of c-myc

Earlier experiments showed that retinoic acid-mediated up-regulation of c-myc correlated with the extent of active cell death of clone

**Figure 4** Anti-TNF-α antibody inhibits AF-mediated apoptosis. Conditioned supernatant was applied to N.1 cells in the presence of 10 μg ml$^{-1}$ non-immune mouse IgG1 (**A**), 0.5 μg ml$^{-1}$ and 2.0 μg ml$^{-1}$ of monoclonal inhibitory anti-TNF-α antibody (**B** and **C** respectively). Photos were taken after 72 h of treatment

N.1 when serum was withdrawn (Krupitza et al, 1995*a*). Also, TNF-α- and AF-triggered apoptosis was preceded by c-myc induction. TNF-induced c-myc stimulation was dose dependent (not shown). Similarly, AF-mediated c-myc expression increased when culture supernatants were conditioned for prolonged time periods which also reflected dose dependence, because over time more AF accumulated in growth media. Non-conditioned supernatants, which were inactive, were utilized for control reasons (Figure 6).

In previous investigations, it was shown that N.1 cells secreted macrophage colony-stimulating factor (M-CSF), which also stimulated c-myc. However, exposure of N.1 cells to increasing concentrations of recombinant M-CSF in combination with 20 ng ml$^{-1}$ recombinant TNF-α neither promoted nor inhibited the apoptotic effect of TNF-α (data not shown).

© Cancer Research Campaign 1998

**Figure 5** Inhibition of AF-induced apoptosis by anti-TNF-α antibody (A.2, A.3, B.2, B.3, C.2). N.1 cells were exposed to conditioned, serum-free ('inducing') supernatant, and for control to non-conditioned, serum-free ('non-inducing') supernatant. The supernatants were preincubated with saline (control), 0.5, 1.0 and 2.0 µg $ml^{-1}$ anti-TNF-α antibody at 37°C for 1 h. After a 72-h exposure of N.1 cells to these preincubated supernatants, the experiments were terminated and apoptosis determined by TUNEL assay. Numbers on the *y*-axis give the percentage of apoptotic cells as mean values of triplicate experiments

**Figure 6** Regulation of mRNA levels in N.1 cells after treatment with conditioned supernatants from progressively confluent N.1 cells. Subconfluent N.1 cultures were treated with supernatant derived from subconfluent cultures (control; lane 1), and with supernatants from confluent and overconfluent cultures (lanes 2 and 3 respectively). Filters were hybridized with c-*myc* probe (**A**), stripped and rehybridized against GAPDH (**B**)

In the presence of 5 µg $ml^{-1}$ and 10 µg $ml^{-1}$ antisense c-myc oligonucleotides (Klefstrom et al, 1994), the activity of AF was significantly (though not completely) inhibited (Figure 7). To prevent oligonucleotide-mediated non-specific cytotoxicity, the serum content was adjusted to 2.5% during experimentation. However, induction of apoptosis took longer under these conditions.

### TNF-α and AF induce plasminogen activator/urokinase (upa) expression

TNF-α up-regulated upa transcript levels in N.1 cells (Figure 8A). The fact that AF also induced upa mRNA with similar kinetics to TNF-α (Figure 8B) further suggested that TNF was a constituent of N.1-conditioned supernatants. Densitometer readings measured the increase in upa transcript levels after stimulation with TNF-α and AF (Table 1).

The time point 1 h after treatment with TNF-α and AF was used as reference point 1. The peak values of the other time points (2, 3, 5, 8 h and control) were set in relation to this reference point, and the numbers shown in Table 1 represent the x-fold expression of upa mRNA. The maxima of upa transcript levels occurred after 2–3 h of induction with TNF-α and AF, and returned to control levels after 8 h of treatment.

Synthesis of uPA protein by N.1 cells was confirmed immunocytochemically (not shown).

### Plasminogen activator/urokinase interferes with cell–matrix contact

N.1 cells were grown on matrigel-coated filters in αMEM containing 10% serum. When cells reached confluency, medium

**Figure 7** Inhibition of AF-induced cell death by antisense *c-myc* oligonucleotides (A.1, C.3). In presence of 2.5% FCS, N.1 cells were incubated with conditioned (+) and non-conditioned (–) supernatant in presence of saline (control), 5 µg $ml^{-1}$ and 10 µg $ml^{-1}$ antisense *c-myc* (As) and scramble (Scr) oligonucleotides. After 7 days, experiments were terminated and per cent of living cells (numbers given on the *y*-axis) were determined by trypan blue exclusion. The mean values of triplicate experiments are shown was discarded and cells washed twice with prewarmed αMEM without serum. Then, cells were incubated with increasing concentrations of uPA at 37°C. After 4 h, incubation medium was aspirated and the cells floating in the medium were counted. Experiments were done in triplicate. On average, 3.6 × $10^3$ cells were floating under control conditions. Addition of 250 U uPA $ml^{-1}$ did not induce an increase in cell detachment. However, incubation with 500 U $ml^{-1}$ and 1000 U $ml^{-1}$ increased the number of released cells to 7.4 × $10^3$ and 10.8 × $10^3$ cells respectively (Figure 9).

These experiments worked only on matrigel-coated membranes, but not on normal cell culture supports such as Petri dishes. This suggested that uPA exerted its activity on components of the extracellular matrix and explained that no difference in apoptosis was seen when cells, grown on Petri dishes, were exposed to

© Cancer Research Campaign 1998

**Figure 8** Kinetic of mRNA expression of N.1 cells which were exposed to 20 ng ml⁻¹ TNF-α (**A**) and N.1-conditioned supernatants containing autocrine factors (**B**) in the absence of FCS. N.1 cells were treated for 1, 2, 3, 5 and 8 h, lanes 1, 2, 3, 5, 8 respectively. Filters were hybridized against *upa* probe, stripped and reprobed with GAPDH

uPA and TNF-α or to TNF-α alone. When tested for one week on N.1 cells grown on Petri dishes, 1000 U uPA ml⁻¹ (which corresponded to 360 ng ml⁻¹) was non-toxic. Thus, when cells were grown on matrigel, the effect of uPA on N.1 cultures was specific.

### DISCUSSION

Neoplasms result from loss of control upon genes regulating growth and/or death. Tumours of repetitive cycles of regression and recurrence (Egawa et al, 1995; Kaufmann et al, 1995) and also lasting spontaneous remissions were observed (Waring et al, 1996). Such processes result from the elimination of transformed cell pools, and these cells either die necrotically or they are phagocytosed by neighbouring or specialized cells of the immune system after apoptosis. This requires fully functional apoptotic machinery which still responds to physiological signals.

**Figure 9** *uPA*-mediated cell detachment from extracellular matrix. N.1 cells were grown on matrigel-coated membranes, which allowed macromolecular access not only from the apical site but also from the basal cellular attachment site. Cells were exposed to 250, 500 and 1000 units *uPA* ml⁻¹ at 37°C, and after 4 h of incubation the number of detached cells was determined. Controls were treated with 1 µg ml⁻¹ BSA. Results are mean values of triplicate experiments

In this investigation, the human ovarian adenocarcinoma model cell line N.1 was used to study mechanisms of self-triggered, physiological apoptosis. Clone N.1 was derived from a single cell (Grunt at al, 1991). When serum was deprived, N.1 cells assumed a more heterogeneous, flattened and enlarged phenotype and started to produce filaments, which suggests that the morphology was dependent on serum factors. The phenotype of cells growing in the middle of a colony was less affected than the phenotype of cells growing at the colony margins. Because margin cells expanded, thereby refilling the gaps between the colonies, the appearance of clone N.1 lost homogeneity.

The results obtained indicate that:

- TNF-α and autocrine factors (AF) induce active cell death.
- N.1 cells express TNF-α and TNF-receptor 1.
- Anti-TNF-α antibody inhibits AF-induced apoptosis of N.1 cells.
- AF-induced apoptosis has some dependence on c-myc expression.
- TNF-α and AF-mediated induction kinetics of upa mRNA transcription are similar.
- Treatment with purified uPA protein interferes with the contact of N.1 cells to the extracellular matrix.

Our results show that TNF and AF triggered apoptosis in N.1 cells. The observation that N.1 cells expressed TNF-α and TNF receptor 1 (and also TNF-β and TNF receptor 2 – not shown) raised the possibility that TNF-α was a constituent of AF, which induced active cell death. This was made evident by ELISA analysis specific against human TNF-α, which detected 48 ng ml⁻¹ TNF-α in serum-free supernatants conditioned by N.1 cells for 10 days. These data are in good correlation with the results obtained by Naylor et al (1993) and Wu et al (1993), who showed that few human ovarian carcinomas produce TNF-α.

© Cancer Research Campaign 1998

Because anti-TNF-α antibody significantly inhibited AF-triggered cell death, TNF-α, which was demonstrated to be synthesized by N.1 cells, contributed to N.1 autocrine apoptosis. Conditioned supernatant, which was adjusted to 20 ng ml$^{-1}$ of secreted TNF-α, induced apoptosis of N.1 cells faster than 20 ng ml$^{-1}$ of recombinant TNF-α in serum-free, non-conditioned medium. This could have been due either to loss of activity of the commercial brand or to the presence of further apoptosis-promoting secreted factors such as TNF-β. A multitude of human recombinant cytokines and growth factors tested in our experiments (IL-1α, IL-1β, IL-2, IL-3, IL-4, IL-6, IL-10, IL-11, SCF, aFGF, IGF-1, IGF-2, insulin, M-CSF, oncostatin, RANTES, angiogenin, LIF, EPO, INF-γ and transferrin) failed to induce apoptosis in N.1 cells.

Like N.1, the slowly growing sister cell line D.3 (Grunt et al. 1991) responded to AF (Krupitza et al. 1995*a*), but did not produce TNF-α itself at the time points investigated. TNF-α secreted by N.1 accumulated late during conditioning of serum-free medium. Immunocytochemistry, however, confirmed that TNF-α was already synthesized by subconfluent cells. Thus, shedding of TNF-α seemed to be a late event.

Despite an accumulation of TNF-α to 48 ng ml$^{-1}$ after 10 days, N.1 cells which were used for conditioning did not undergo apoptosis. Only N.1 cells that were kept in frequently changed culture medium, or which were subconfluent, maintained their responsiveness to AF and TNF-α. This suggests that a negative feedback mechanism protected old-growth N.1 cultures from the effects of a TNF–TNF receptor circuit.

TNF-α-induced apoptosis in Rat1A-MycERcells, NIH3T3-cMyc cells and WEHI 164/13S mouse fibrosarcoma cells was shown to be c-myc dependent (Klefstrom et al. 1994). In an earlier investigation, we have shown that N.1 cells secreted M-CSF, which also stimulated c-myc (Krupitza et al. 1995*a*), and that c-myc induction correlated with retinoic acid-induced apoptosis (Krupitza et al. 1995*b*). However, at low serum levels, the administration of recombinant M-CSF alone or in combination with recombinant TNF-α did not induce or support apoptosis respectively.

Therefore, the induction of c-myc itself did not induce apoptosis in N.1 cells even when serum was withdrawn, but it seemed that it depended on the physiological ligand by which c-myc was induced. Either ligands such as M-CSF, which stimulated c-myc but did not trigger apoptosis, induced additional pathways ensuring survival. Alternatively, induced c-Myc might have become modified to transcribe certain genes upon M-CSF treatment, whereas distinct targets were influenced by c-Myc upon treatment with TNF-α.

Incubating N.1 cells, which were exposed to AF, with 5 and 10 µg ml$^{-1}$ antisense c-myc oligonucleotides showed specific inhibition of apoptosis. Thus, although M-CSF-mediated stimulation of c-myc did not induce apoptosis in N.1 cells, AF-induced apoptosis of N.1 cells was in part c-myc dependent.

Apoptotic stimuli were enforced when cell contact to the growth substrate was disrupted. Cell–matrix attachment provides significant endogenous survival signals, and dissolution of cell–matrix interactions by protease supports apoptotic stimuli (Frisch and Francis, 1994). TNF-α and AF up-regulated transcription of plasminogen activator/urokinase (upa) with similar kinetics. We have shown that purified plasminogen activator disrupted cell–matrix interactions in the N.1 model system in a dose-dependent manner. Consequently, in a physiological situation, upa might contribute to TNF-induced effects by facilitating an anoikis type of cell death (enhanced apoptotic response due to loss of cell-matrix interactions; Frisch and Francis, 1994). UPA, secreted for example by keratinocytes, elicits a protease cascade leading to activated plasmin (Bechtel et al. 1996). It was reported that uPA colocalizes pericellularly with the αvβ5 vitronectin receptor subtype. In its proximity, the extracellular matrix protein vitronectin is degraded and keratinocytes lose cell matrix contact (Reinartz et al. 1995), thereby exposing the vitronectin receptor. Exposition of vitronectin receptor subtype αvβ3 was shown to provide an 'eat me' signal for macrophages (Fadok et al. 1992). Therefore, uPA plays a role in cell elimination processes.

However, apoptosis of N.1 cells did not depend on uPA activity because N.1 cells underwent cell death upon TNF-α and AF treatment when grown on normal cell culture dishes that were not coated with extracellular matrix proteins and at which uPA could not exert an apoptosis supporting effect. Thus, the apoptotic effect of AF is direct and not triggered by uPA-mediated matrix dissolution. Nevertheless upa overstimulation might contribute to cell death in a physiological context by disrupting cell–matrix attachment.

We conclude that N.1 cells are capable of autocrine self-elimination by a TNF–TNF-receptor circuit. To test whether this is a relevant mechanism in tumour cell biology, different TNF-producing tumour cell lines need to be investigated as to their potential to trigger self destruction. Searching for factors that rescue TNF-α-stimulated apoptosis as well as factors specifically regulating the expression of TNF receptors would provide further insight and would allow for the strict regulation of this apoptosis mechanism.

### ACKNOWLEDGEMENTS

We want to acknowledge Isabella Mosberger and Andrea Lamprecht for excellent technical assistance, Anika Krupitza for preparing parts of the figures, Dr Christoph Kopp for helpful discussion and Dr Andreas Koeck for generously providing an ELISA Anthos-reader. This work was supported by the 'Kommission Onkologie' of the Medical Faculty of Vienna.

### REFERENCES

Askew D, Ashmun R, Simmons B and Cleveland J (1991) Constitutive c-myc expression in an IL-3 dependent myeloid cell line suppresses cell cycle arrest and accelerates apoptosis. *Oncogene* **6**: 1915–1922

Bechtel M, Reinartz J, Ros J, Inndorf S, Schaefer B and Kramer M (1996) Upregulation of cell-surface-associated plasminogen activation in cultured keratinocytes by interleukin-1 beta and tumour necrosis factor alpha. *Exp Cell Res* **223**: 395–404

Buick R, Pullano P and Trent J (1985) Comparative properties of five human ovarian adenocarcinoma cell lines. *Cancer Res* **45**: 3668–3676

Delbaldo C, Cunningham M, Vassalli J and Sappino A (1995) Plasmin-catalyzed proteolysis in colorectal neoplasia. *Cancer Res* **55**: 4688–4695

Egawa N, Fukayama M, Kawaguchi K, Hishima T, Hayashi Y, Funata N, Ibuka T, Koike M, Miyashita H and Tajima T (1995) Relapsing oral and colonic ulcers with monoclonal T-cell infiltration: a low grade mucosal lymphoproliferative disease of the digestive tract. *Cancer* **75**: 1728–1733

Evan G, Wyllie A, Gilbert C, Littlewood T, Land H, Brooks M, Waters C and Hancock D (1992) Induction of apoptosis in fibroblasts by c-myc protein. *Cell* **69**: 119–128

Fadok V, Savill J, Haslett C, Bratton D, Doherty D, Campbell P and Henson P (1992) Different populations of macrophages use either the vitronectin receptor or the phosphatidylserine receptor to recognize and remove apoptotic cells. *J Immunol* **149**: 4029–4035

Frisch S and Francis H (1994) Disruption of epithelial cell–matrix interactions induces apoptosis. *J Cell Biol* **124**: 619–626

© Cancer Research Campaign 1998 

Grunt T. Dittrich E. Somay C. Wagner T and Dittrich C (1991) Separation of clonogenic and differentiated cell phenotypes of ovarian cancer cells HOC-7 by discontinuous density gradient centrifugation. *Cancer Lett* **68**: 7–16

Janicke R. Lee F and Porter A (1994) Nuclear c-myc plays an important role in the cytotoxicity of tumor necrosis factor alpha in tumor cells. *Mol Cell Biol* **14**: 5661–5670

Kaufmann Y. Many A. Rechavy G. Mor O. Bimianimov M. Rosenthal E. Levanon M. Davidson J. Aizman I. Mark Z. Brok-Simoni F and Ramot B (1995) Brief report: lymphoma with recurrent cycles of spontaneous remission and relapse – possible role of apoptosis. *N Engl J Med* **332**: 507–510

Klefstrom J. Vastrik I. Saksela E. Valle J. Eilers M. Alitalo K (1994) C-Myc induces cellular susceptibility to the cytotoxic action of TNF-alpha. *EMBO J* **13**: 5442–5450

Krupitza G. Fritsche R. Dittrich E. Harant H. Huber H. Grunt T and Dittrich C (1995*a*). Macrophage colony-stimulating factor is expressed by an ovarian carcinoma subline and stimulates the c-myc proto-oncogene. *Br J Cancer* **72**: 35–40

Krupitza G. Hulla W. Harant H. Dittrich E. Kallay E. Huber H. Grunt T and Dittrich C (1995*b*) Retinoic acid induced death of ovarian carcinoma cells correlates with c-myc stimulation. *Int J Cancer* **61**: 649–657

Meichle A. Schutze S. Hensel G. Brunsig D and Kronke M (1990) Protein kinase C-independent activation of nuclear factor kappa B by tumour necrosis factor. *J Biol Chem* **265**: 8339–8343

Naylor M. Stamp G. Foulkes W. Eccles D and Balkwill F (1993) Tumor necrosis factor and its receptors in human ovarian cancer. *J Clin Invest* **91**: 2194–2206

Novak U. Cocks B and Hamilton J (1991) A labile repressor acts through the NFkB-like binding sites of the human urokinase gene. *Nucleic Acids Res* **19**: 3389–3393

Reinartz J. Schaefer B. Batrla R. Klein C and Kramer M (1995) Plasmin abrogates alpha v beta 5-mediated adhesion of human keratinocyte cell line (HaCat) to vitronectin. *Exp Cell Res* **220**: 274–282

Simonitsch I. Panzer-Gruemayer E. Ghali D. Zoubek A. Radaszkiewicz T. Gadner H and Kovar H (1996) NPM/ALK gene fusion transcripts identify a distinct subgroup of null type Ki-1 positive anaplastic large cell lymphomas. *Br J Haematol* **92**: 866–871

Waring A. Takata M. Rehman I and Rees J (1996) Loss of heterozygosity analysis of keratoacanthoma reveals multiple differences from cutaneous squamous cell carcinoma. *Br J Cancer* **73**: 649–653

Wu S. Boyer C. Whitaker R. Berchuck A. Wiener J. Weinberg J and Bast R Jr (1993) Tumor necrosis factor α as an autocrine and paracrine growth factor for ovarian cancer: monokine induction of tumor cell proliferation and tumor necrosis factor α expression. *Cancer Res* **53**: 1939–1944

 © Cancer Research Campaign 1998